# Village Homes

**Published:** 1888-11-17

**Authors:** Agnes Wainewright

**Affiliations:** Ken Rectory, Wickham Market


### Editor's Letter-Box.

[Our Correspondents are reminded that prolixity is a great bar to publication, and that brevity of style and conciseness of statement greatly facilitate early insertion.]

### VILLAGE HOMES.

I understand that several matrons write to you to find homes for children and aged people; some of our parishioners would be glad to undertake this. Our village is a purely agricultural one, and very healthy; it is only four miles from the sea. Agnes Wainewright.

Ken Rectory, Wickham Market.

